# Temporomandibular joint (TMJ) morphology in jaw deformity patients (class II and III) before and after surgical orthodontic treatment: a narrative review

**DOI:** 10.1186/s40902-026-00512-0

**Published:** 2026-05-08

**Authors:** Koichiro Ueki, karen gomi, kohara Riku, Yusuke Kurosawa, Kunio Yoshizawa, Akinori Moroi

**Affiliations:** Department of Oral and Maxillofacial Surgery, Division of Medicine, Interdisciplinary Graduate School, University of Yamanashi, Chuo, Japan

**Keywords:** Jaw deformity, Occlusion, Orthognathic surgery, Temporomandibular joint, Morphology

## Abstract

Understanding changes in temporomandibular joint (TMJ) and ramus morphology in skeletal Class II and Class III jaw deformity patients before and after surgical orthodontic treatment is essential for achieving stable postoperative outcomes. Alterations in the positional relationship and morphology of the condyle, TMJ disc, and glenoid fossa can significantly influence postoperative occlusion and skeletal stability. Previous studies have demonstrated close associations among maxillofacial morphology, occlusion, and TMJ structure.

In this narrative review, we summarize findings from our previous investigations regarding changes in TMJ morphology—including disc position, joint space, macroscopic morphology of the condyle and ramus, and computed tomography (CT) values—as well as considerations based on stress analysis in skeletal Class II and Class III deformities. These insights may contribute to a better understanding of anatomical characteristics of the TMJ in jaw deformity patients and may aid in predicting postoperative morphological changes.

## Introduction

Sagittal split ramus osteotomy (SSRO) is one of the most commonly used mandibular osteotomies and is frequently combined with Le Fort I osteotomy in orthognathic surgery [1]. Postoperative changes in condylar position may lead to malocclusion, early relapse, and an increased risk of temporomandibular disorders (TMD) [2–6]. To address these concerns, various condylar positioning devices have been developed; however, neither mandibular advancement nor setback surgery consistently provides stable outcomes [7]. These devices are based on the assumption that the condyle can be repositioned to its exact preoperative three‑dimensional location [8]. Nevertheless, previous reviews have questioned whether the postoperative condylar position truly matches the preoperative position [9], and another review concluded that there is no scientific evidence supporting the contribution of such devices to postoperative occlusal or skeletal stability [10].

Orthognathic surgery involves repositioning the maxillomandibular skeleton, which inevitably alters muscle vectors and occlusal force distribution. These changes may influence postoperative blood supply to the proximal segment and modify stress distribution within the temporomandibular joint (TMJ), potentially leading to morphological changes. Furthermore, many patients present with pre‑existing TMJ internal derangement, yet these pathological factors are not accounted for in the concept of condylar positioning devices.

Progressive condylar resorption (PCR) is characterized by pathological structural changes of the condyle, including reductions in condylar height or volume [11]. PCR is particularly associated with postoperative instability in skeletal Class II patients undergoing mandibular advancement [12–14]. Although its etiology remains unclear, PCR has been linked to female sex, age 20–30 years, high mandibular plane angle, TMJ pathology, excessive mandibular advancement, and counterclockwise rotation of the proximal segment during SSRO [13, 15].

PCR was initially reported in high‑angle Class II patients with mandibular hypoplasia and open bite following bimaxillary osteotomy [16, 17]. Although SSRO was historically considered the primary cause, PCR has also been observed after isolated Le Fort I osteotomy [11, 12]. Clinically, PCR is often recognized only after the development of postoperative anterior open bite or when TMJ abnormalities become evident on radiographs, CT, or MRI. Macroscopic and structural changes of the condyle reflect mechanical loading and the adaptive capacity of the TMJ [18, 19]. However, PCR remains poorly defined, and the lack of quantitative criteria makes it difficult to distinguish between condylar resorption and remodeling.

In TMJ osteoarthritis (OA), disc displacement and degenerative changes lead to subchondral bone erosion, osteophyte formation, fibrocartilage loss, and synovitis. Mechanical loading can induce osteochondral changes that contribute to joint degeneration [20, 21]. Although the role of subchondral bone in OA pathogenesis is not fully understood, condylar resorption may represent part of the degenerative process.

Only a limited number of studies have quantitatively evaluated reductions in condylar height using CT or MRI [22–24]. In cases where postoperative open bite or TMJ pathology obscures the PCR process, reductions in condylar height or volume may go unnoticed. Moreover, it remains unclear whether condylar resorption occurs in Class III patients following bimaxillary osteotomy.

The aim of this narrative review is to summarize current evidence regarding changes in TMJ morphology in skeletal Class II and Class III patients before and after orthognathic surgery, based on CT and MRI findings.

### Literature search strategy

Although this review was conducted as a narrative synthesis, a structured approach was adopted to identify relevant studies. Searches were performed in PubMed and Scopus using combinations of the following keywords: temporomandibular joint, orthognathic surgery, Class II, Class III, condylar resorption, disc displacement, and TMJ morphology.

A simplified PICO framework guided study selection:

- Population: skeletal Class II and III jaw‑deformity patients
- Intervention: orthognathic surgery (SSRO, IVRO, Le Fort I)
- Comparison: pre‑ vs. postoperative CT/MRI findings
- Outcome: TMJ disc position, condylar morphology, glenoid fossa morphology, CT values

Studies were included if they reported imaging‑based assessments of TMJ morphology before and after surgery. Case reports and studies without imaging data were excluded.

## TMJ disc position

The normal temporomandibular joint (TMJ) disc is a biconcave fibrocartilaginous structure positioned between the 11:30 and 12:30 o’clock positions on the condyle [25]. The posterior band is typically thicker than the anterior band, and the medial portion is thicker than the lateral portion, which helps stabilize the disc over the condylar head [26]. In a normal joint, the posterior band is located superior to the condyle in the closed‑mouth position [27–31]. Although the normal disc position has been described in previous studies, these definitions did not consider skeletal morphology or occlusal patterns [32, 33]. In clinical practice, disc morphology and position vary considerably across different skeletal and occlusal types.

Previous studies have shown that anterior disc displacement (ADD) is more prevalent in Class II and Class II open‑bite patients than in Class I or Class III patients, suggesting that ADD may influence mandibular and maxillofacial development [34–40].

In contrast, TMJ morphology in Class III patients can be categorized into four disc types:

1. ADD,
2. anterior type,
3. fully covered type, and
4. posterior type.

**Fig. 1:**
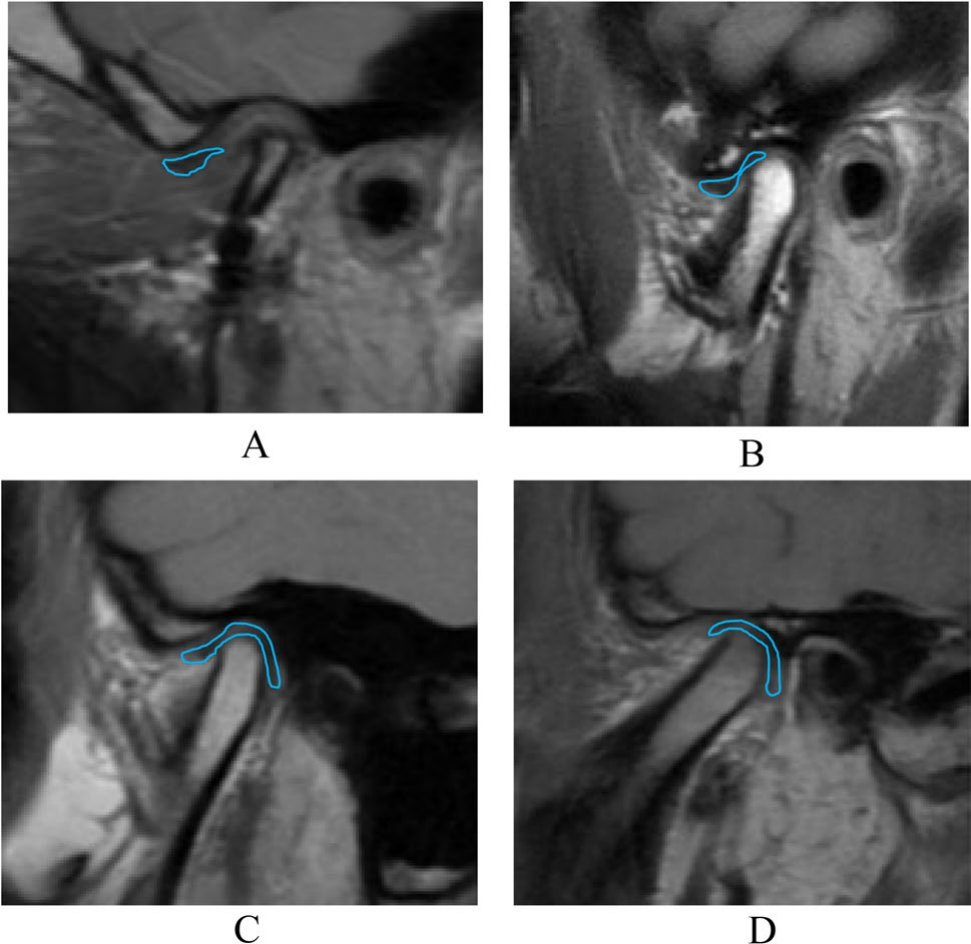
Classification of temporomandibular joint (TMJ) disc position on magnetic resonance imaging. **A** anterior disc displacement with or without reduction; **B** anterior type; **C** fully covered type; **D** posterior type. Red lines indicate the TMJ disc

The fully covered and posterior types are characteristic of Class III morphology [41] (Fig. 1). In asymmetric Class III patients, ADD is more common on the deviated side, whereas the posterior type is more common on the non‑deviated side [41, 42].

**Fig. 2:**
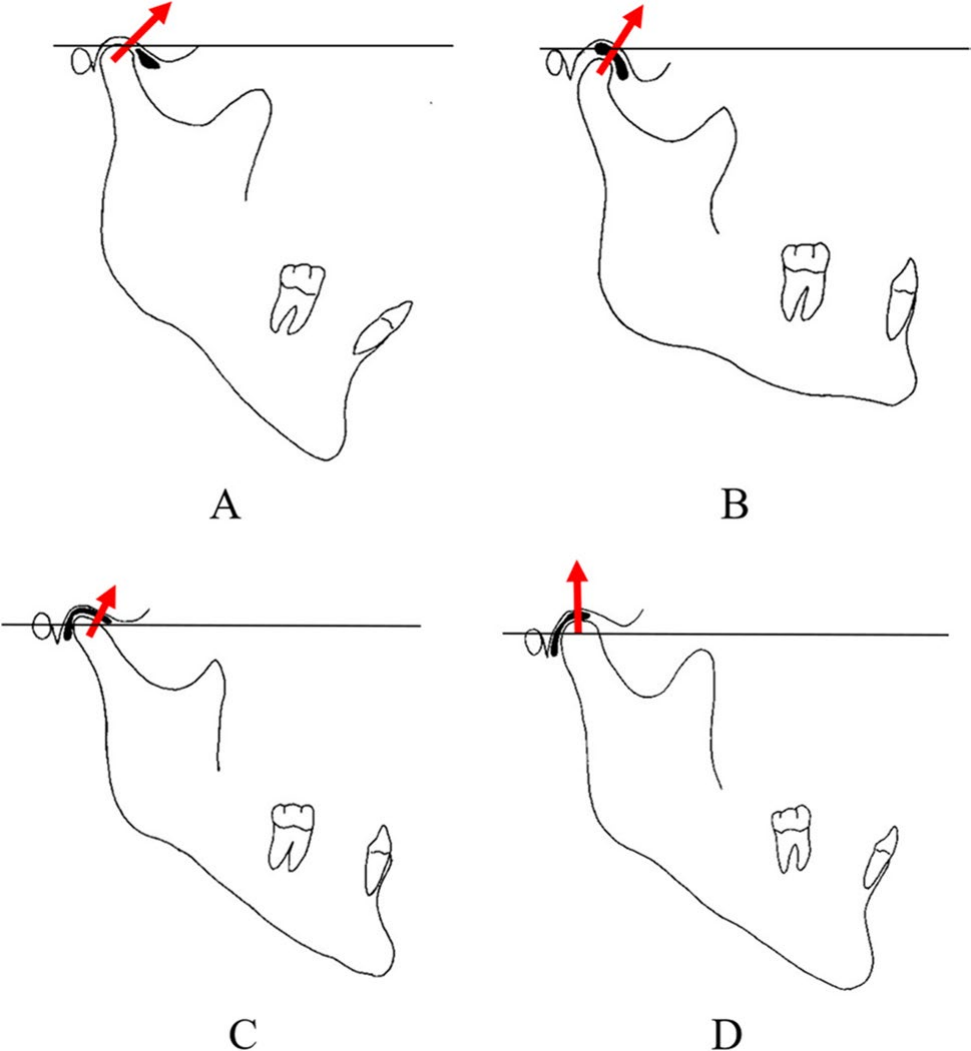
Relationship between disc position and stress direction on the mandibular condyle based on dynamic analysis. **A** anterior displacement with or without reduction; **B** anterior type; **C** fully covered type; **D** posterior type

Using a rigid body spring model (RBSM), previous studies demonstrated that TMJ stress direction strongly correlates with disc morphology in Class III patients with and without asymmetry [43]. In ADD or anterior‑type joints, stress is directed more anteriorly on the condyle, whereas in fully covered or posterior‑type joints, stress is directed more superiorly [43] (Fig. 2).

**Fig. 3:**
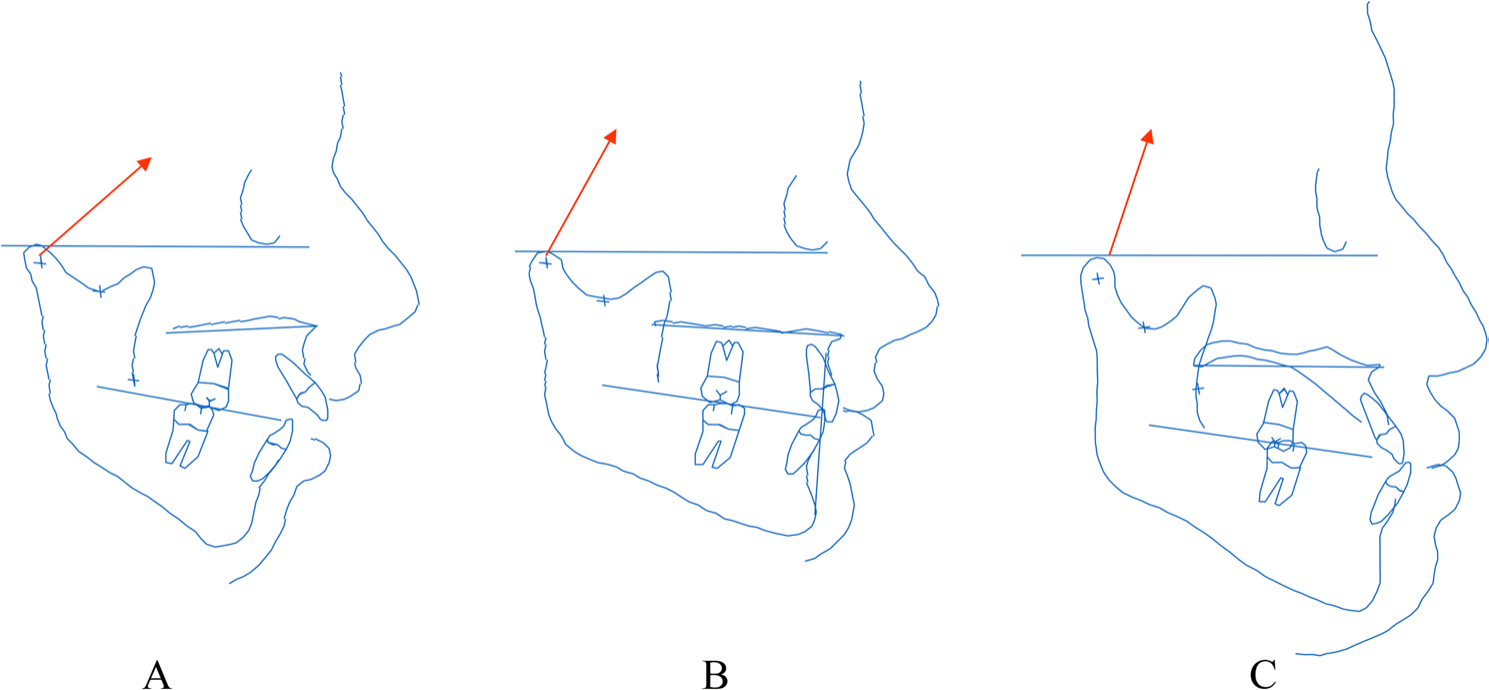
Schematic representation of stress direction on the mandibular condyle based on skeletal morphology. **A** Class II; **B** Class I; **C** Class III. In Class II, stress tends to be directed anteriorly; in Class III, stress tends to be directed superiorly

**Fig. 4:**
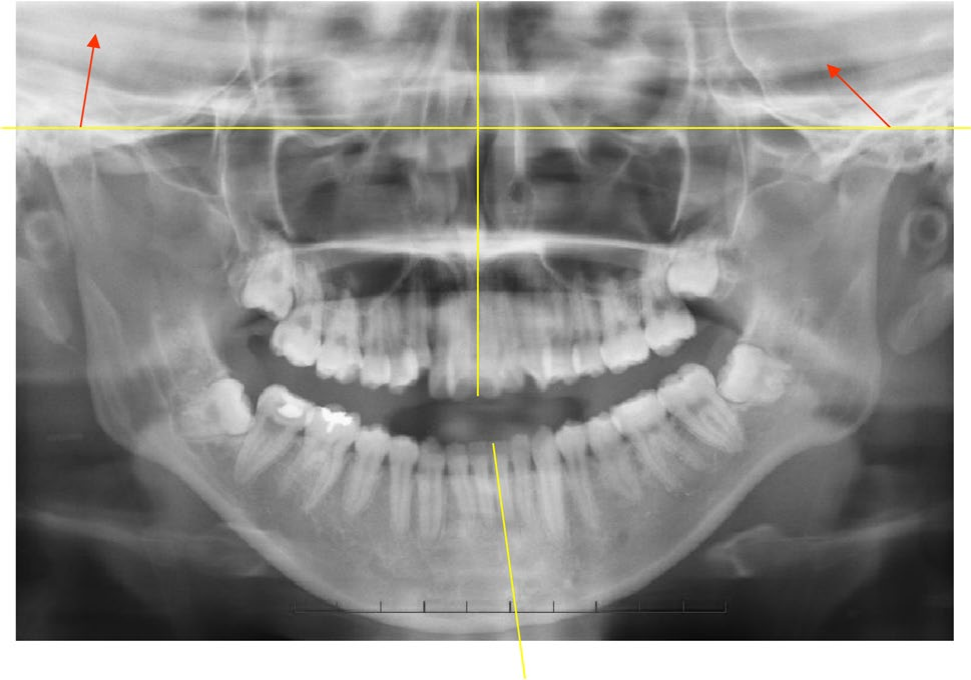
Correlation between right–left differences in condylar stress direction and the degree of mandibular asymmetry

A comparative study among skeletal classes further showed that Class II patients exhibit a more anteriorly directed condylar stress vector than Class I or Class III patients [44] (Fig. 3). Additionally, the right–left difference in stress direction correlates with the degree of mandibular asymmetry [43] (Fig. 4). These findings support a mechanical model in which disc position adapts to the direction of functional loading: anteriorly in Class II and on the deviated side of mandibular asymmetry.

These insights also help clarify the etiology of posterior disc displacement (PDD), a rare condition in TMD patients [45]. Reported PDD morphologies include:

1. a thin disc extending posteriorly,
2. a perforated disc with a small anterior and large posterior component, and
3. a disc entirely posterior to the condyle [46].

Other studies have described thin, flat, or perforated discs but rarely true posterior displacement [47]. A systematic review concluded that PDD is extremely uncommon and its characteristics remain unclear [48].

Some reports suggested that PDD may cause a posterior open bite on the affected side without midline deviation [49], and may be associated with pain, joint sounds, open lock, or TMJ dislocation [47]. However, these studies may have misinterpreted disc position. They described cases in which the disc appeared normal in the closed‑mouth position but shifted posteriorly during mouth opening [47, 49]. This likely reflects normal movement of the temporal posterior attachment (TPA), which appears as a thin band on MRI in the closed‑mouth position.

True PDD must be diagnosed in the closed‑mouth, occluded position [50–53]. Many previously reported “PDD” cases likely correspond to the posterior or fully covered types commonly observed in Class III patients and on the non‑deviated side of mandibular asymmetry.

TMJ dislocation may be associated with abnormalities of the condyle, articular eminence, disc, ligaments, or masticatory muscles [47]. In Class III patients with posterior‑type discs, these factors may increase susceptibility to TMJ luxation. Supporting this, Class II patients show greater condylar movement than Class I patients [54], whereas Class III patients exhibit flatter condylar paths during protrusion and mediotrusion, and a smaller curvature of the condylar path compared with Class II patients [54]. Class III patients also tend to have shallower glenoid fossa [55], which may facilitate anterior dislocation.

MRI studies of asymptomatic individuals have shown disc displacement in approximately one‑third of joints [56, 57], suggesting that asymptomatic ADD in Class II patients may represent an anatomical variant rather than a pathological condition [58, 59]. Similarly, posterior‑type and fully covered discs in Class III patients may represent normal anatomical adaptations.

Chewing‑path studies have shown that Class III patients exhibit a linear, chopping‑type movement with large vertical opening, whereas non‑prognathic patients show lateral grinding movements [60, 61]. Class III patients with symmetry maintain this linear pattern, whereas asymmetric Class III patients do not [62]. Because Class III patients have a long mandibular body and can achieve wide opening with minimal condylar translation, disc mobility is less critical, and posterior disc position does not impair function. This contrasts with Class I and II patients, in whom a high articular eminence and ADD may impede reduction during dislocation.

Previous studies have shown that the prevalence of ADD (with or without reduction) is high in Class II patients [55, 63–65]. One year after orthognathic surgery, disc classification generally remains unchanged in both Class II and Class III patients. Our studies demonstrated that SSRO, with or without Le Fort I osteotomy, does not alter preoperative disc position, including ADD. In contrast, intraoral vertical ramus osteotomy (IVRO) can improve ADD and TMJ symptoms due to temporary condylar sag caused by lateral pterygoid traction immediately after surgery [66–68]. However, the improvement rate is low, and the postoperative condylar position differs markedly from the preoperative position.

Other studies have reported that ADD rarely improves after SSRO advancement in Class II patients. Improvements observed on postoperative MRI may be questionable, particularly in ADD without reduction, which does not typically normalize after surgery. Some ADD with reduction may convert to ADD without reduction, and some normal discs may develop ADD postoperatively [69–73]. In Class III setback surgery, disc classification generally remains unchanged, although minor changes in disc length have been reported [74–76].

Overall, disc position—including ADD, anterior type, fully covered type, and posterior type—remains stable after orthognathic surgery in both Class II and Class III patients, except in cases treated with IVRO. Several MRI‑based studies have demonstrated that anterior disc displacement (ADD) typically remains unchanged after SSRO, even in cases involving mandibular advancement [41, 55, 63–67, 77, 78]. Improvements reported in some studies may reflect methodological differences or misinterpretation of the posterior attachment [69]. Consistent with previous reports, disc classification is generally stable postoperatively, except in cases treated with intraoral vertical ramus osteotomy (IVRO) [42, 66, 68].

The timeline of postoperative changes varies depending on the surgical technique. Most imaging‑based evaluations in the literature were performed 6–12 months after surgery, when early remodeling has stabilized. After 1 year, SSRO generally does not alter preoperative disc position, whereas IVRO may temporarily improve disc position due to condylar sag induced by lateral pterygoid traction [41, 42, 55, 63–68, 77, 78]. However, this improvement is often partial and unpredictable, and postoperative condylar position differs substantially from the preoperative state.

## Condyle and glenoid fossa morphology

In this review, progressive condylar resorption (PCR) refers to pathological structural changes of the mandibular condyle characterized by: condylar height reduction exceeding 2 mm, decrease in condylar volume on 3D imaging, and/or radiographic evidence of cortical erosion, subcortical sclerosis, or osteolytic changes on CT or MRI.

These criteria reflect commonly used diagnostic parameters in previous studies.

Progressive condylar resorption (PCR) has traditionally been evaluated primarily in Class II patients [13, 15, 18, 19, 79–82]. However, recent studies indicate that reductions in condylar volume may occur not only in Class II but also in Class III patients, although the degree of reduction is significantly greater in Class II cases [55, 63–65]. TMJs with anterior disc displacement (ADD) show a higher rate of condylar height reduction than joints with other disc positions. In one study, a Class III patient also exhibited PCR, but the affected joint had ADD [63], suggesting that disc displacement may be a more critical factor than skeletal classification alone.

Cone‑beam CT (CBCT) has been widely used to evaluate condylar morphology and positional changes after orthognathic surgery [83, 84]. A quantitative 3D CBCT analysis in Class III patients demonstrated postoperative bone formation predominantly in the anteromedial region of the condyle and bone resorption in the anterolateral region following SSRO [85]. Another study reported corresponding changes in the glenoid fossa, with bone formation in the anterolateral fossa and bone resorption in the anterolateral condyle [86].

In Class II mandibular advancement cases, condylar resorption was observed in approximately one‑third of joints, and the glenoid fossa cavity volume increased significantly after surgery, although it gradually returned toward preoperative levels over time [87]. Another study found that glenoid fossa remodeling was more pronounced in joints with condylar resorption than in those without, particularly in the anterolateral and anteromedial regions [88]. Additional research has confirmed postoperative adaptive changes in both the condyle and glenoid fossa in Class II patients [89].

Our previous work demonstrated decreases in condylar height and ramus height after surgery in both Class II and Class III patients. In Class II patients, the sagittal area of the glenoid fossa increased after bimaxillary surgery [55]. These findings suggest that postoperative macroscopic morphological changes occur in both the condyle and the glenoid fossa, regardless of skeletal classification.

In Class III patients, postoperative remodeling patterns differ from those observed in Class II cases. Class III patients often present with fully covered or posterior disc types, which may represent adaptive responses to superiorly directed joint loading. These characteristics may contribute to the relatively lower incidence of PCR in Class III patients compared with Class II patients, despite the presence of postoperative remodeling [55, 65, 78].

## Surface CT values in condyle and glenoid fossa

To improve clarity, terminology has been standardized throughout the manuscript:

- Condylar resorption refers to pathological loss of condylar bone.
- Condylar height reduction refers to linear dimensional decrease.
- Condylar volume reduction refers to 3D volumetric loss.

Osteoarthritis (OA) of the temporomandibular joint (TMJ), with or without symptoms, is one of the major pathological conditions affecting the joint. According to the Research Diagnostic Criteria for Temporomandibular Disorders (RDC/TMD), CT is recommended for evaluating osseous components, while MRI is used to assess disc position [26]. Disc displacement is included as a non‑osseous diagnostic criterion for OA, and subcortical sclerosis is considered a key radiographic feature [26]. Previous studies have reported OA prevalence rates of 3% in Class I, 43% in Class II, and 20% in Class III patients [90]. However, these diagnostic criteria are qualitative and lack quantitative definitions.

For this reason, quantitative evaluation of TMJ hard tissues is essential. The most reliable method for assessing the internal structure of the condyle is measurement of the Hounsfield unit (CT value), which correlates with bone density. Bone strength is strongly associated with both bone density and bone quality [91]. CT values are also useful for determining material properties in biomechanical analyses such as finite element modeling. Although CBCT does not provide true CT values, medical CT allows objective assessment of bone density. Because CT values vary across scanners and imaging conditions, standardized imaging protocols are required. The maximum CT value consistently appears in the cortical surface of the condyle and glenoid fossa, making it a reliable and reproducible parameter.

**Fig. 5:**
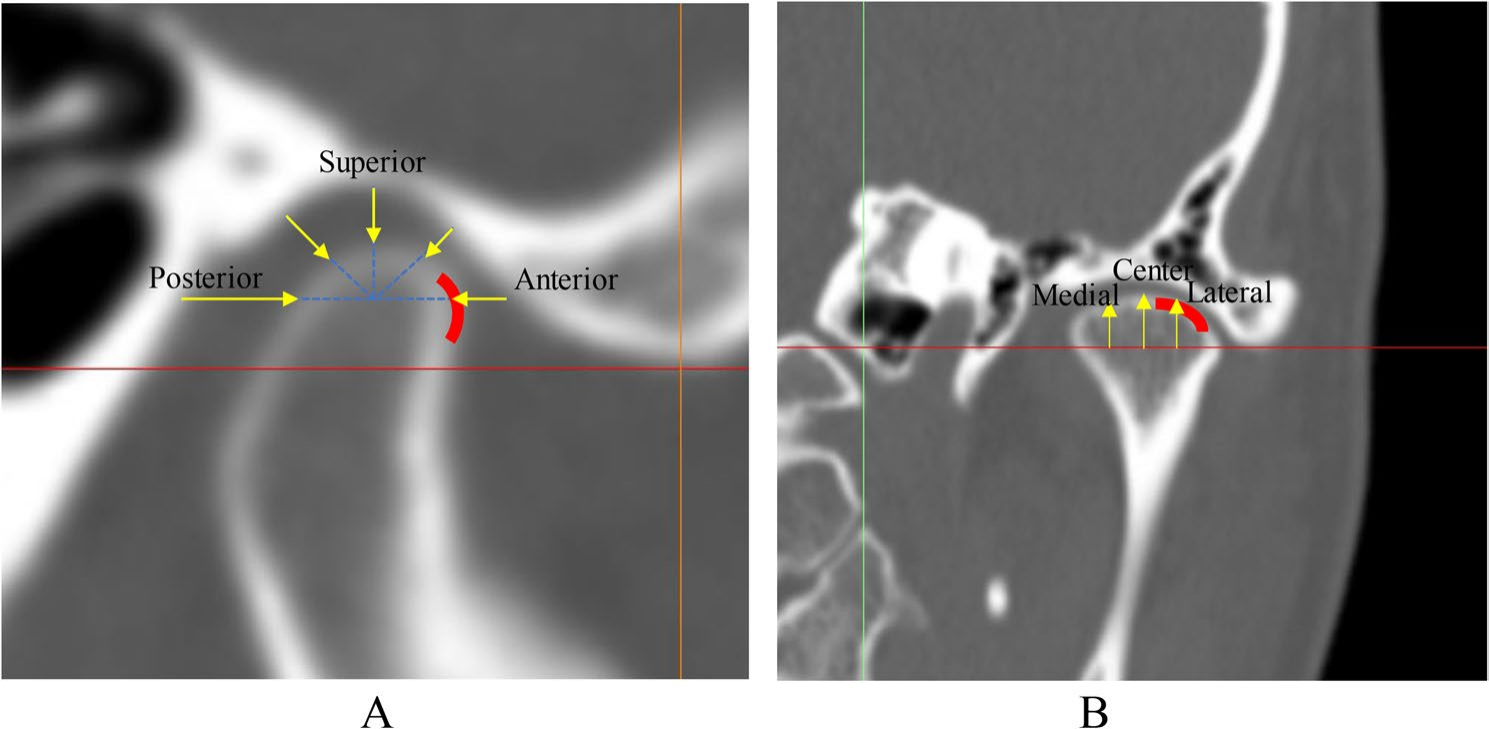
Condylar surface CT values. Red lines indicate regions with higher CT values. **A** Sagittal plane: higher CT values in Class II than Class III; anterior region > posterior region; postoperative (1 year) > preoperative. **B** Coronal plane: higher CT values in Class II than Class III; lateral region > medial region; postoperative (1 year) > preoperative

Previous studies have shown that condylar surface CT values are higher in joints with ADD than in joints with other disc types [77]. CT values are also higher in Class II than in Class III patients. In sagittal plane analyses, Class II patients with ADD exhibit higher CT values anteriorly than posteriorly [65] (Fig. 5A). These findings indicate that CT value distribution differs between skeletal classes and between disc‑position categories. Furthermore, high CT values may contribute to postoperative condylar height reduction, suggesting a link between increased bone density (subcortical sclerosis) and subsequent resorption [69].

Postoperatively, condylar surface CT values increase in Class II patients but remain unchanged in Class III patients. Similar trends are observed in the glenoid fossa [55]. Regions with postoperative narrowing of the TMJ space correspond to areas with high CT values in both the condyle and glenoid fossa. Posterior‑superior displacement of the proximal segment after surgery may alter stress distribution, inducing internal structural changes in both the condyle and glenoid fossa. High CT values in the glenoid fossa may serve a protective role for the skull base and brain; however, increased density may also reflect sclerosis associated with condylar resorption [80, 92–94].

Low bone quality of the condyle has been associated with TMJ OA development, and both CT values and bone volume fraction (BV/TV) have been proposed as diagnostic indicators [95]. However, previous studies evaluated the condyle as a whole and did not examine regional variations. Condylar density likely varies by anatomical region and may differ depending on the stage of OA. Early OA is characterized by increased density (sclerosis), whereas late‑stage OA may show decreased density due to osteolysis. Subcortical sclerosis can rapidly progress to osteolysis when mechanical loading increases or when postoperative nutritional compromise occurs. In our previous study, condylar surface CT values and height reduction were greater in Class II than in Class III patients, whereas glenoid fossa resorption was not observed [55], suggesting different mechanisms of morphological change between the condyle and glenoid fossa.

A coronal CT study demonstrated a negative correlation between condylar surface CT values and condylar height at central and lateral regions, both pre‑ and postoperatively [78] (Fig. 5B). This suggests that higher CT values are associated with reduced condylar height. Additionally, Class II patients exhibit greater horizontal condylar angles than Class III patients both before and after surgery. After SSRO advancement, the condylar angle decreases significantly in Class II patients, likely due to outward rotation of the proximal segment rather than new bone formation [78].

Previous studies have shown that TMJs with ADD or internal derangement exhibit higher horizontal condylar angles than normal joints, and resorption of the lateral pole (RLC) has been proposed as a contributing factor [96, 97]. However, these studies did not report skeletal classification or occlusion, and the findings likely reflect characteristics of Class II patients, who frequently present with ADD and increased horizontal condylar angles. Our findings indicate that higher CT values are associated with lower condylar height and greater horizontal condylar angle [78]. Postoperative increases in lateral CT values in Class II patients may reflect RLC following SSRO. Distinguishing between congenital hypoplasia and postoperative resorption remains challenging.

## A hypothesis regarding the etiology of PCR based on stress analysis and CT values

The etiology of progressive condylar resorption (PCR) can be considered by integrating findings from condylar surface CT values and stress‑distribution analyses of the TMJ [43, 44, 55, 65, 78]. Class II patients with high mandibular plane angles frequently present with anterior disc displacement (ADD) and posteriorly inclined condylar necks. Preoperatively, the direction of condylar stress is oriented more anteriorly, and the primary loading site is located on the anterior aspect of the condyle. In this context, ADD may represent an adaptive mechanism that maintains functional stress balance in the TMJ.

**Fig. 6:**
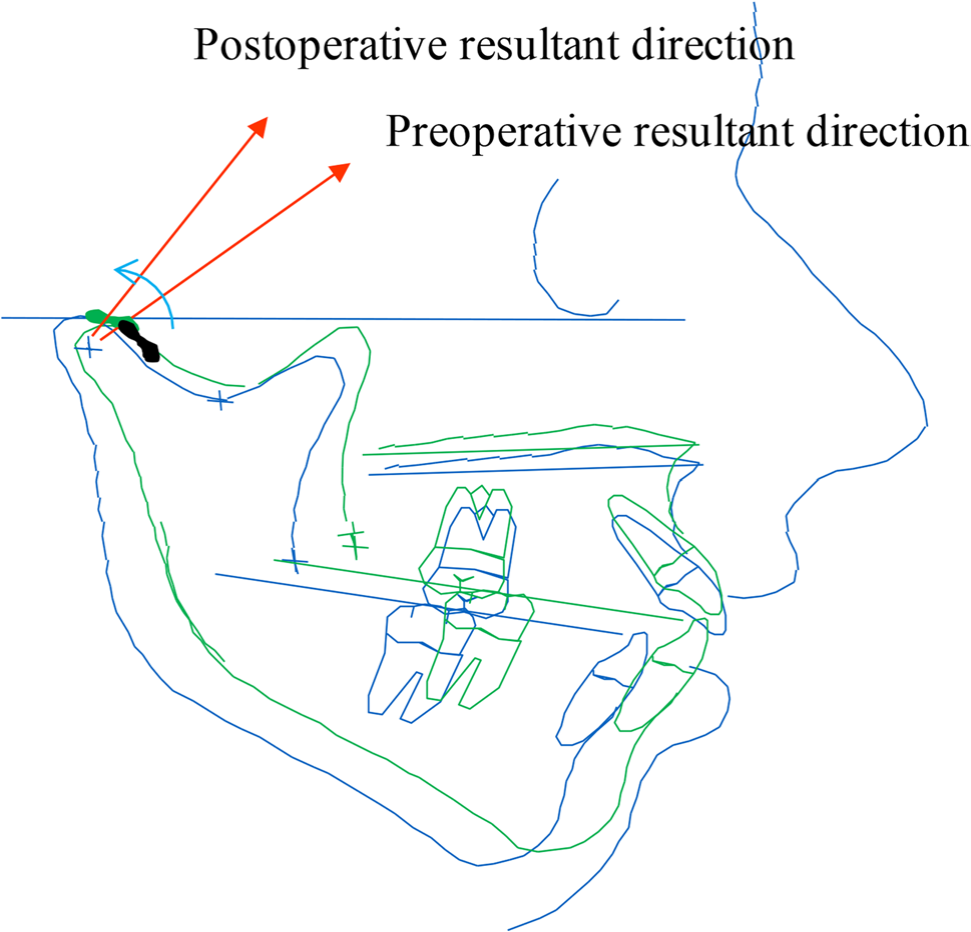
Conceptual model of progressive condylar resorption (PCR). Counterclockwise rotation and mandibular advancement shift the stress‑bearing site superiorly, while the anteriorly displaced disc remains unchanged. In Class II open‑bite patients with anterior disc displacement, the anterior condylar surface often exhibits high density (high CT value)

After SSRO with mandibular advancement, counterclockwise rotation and posterior displacement of the proximal segment commonly occur. As a result, the primary loading site shifts superiorly. However, the anteriorly displaced disc often remains in its preoperative position. This mismatch between the new loading site and the unchanged disc position may generate concentrated stress on an uncovered region of the anterior condylar surface (Fig. 6). These regions may be more susceptible to localized remodeling or resorptive changes, although the precise threshold of mechanical loading required to induce PCR remains unclear.

Following mandibular osteotomy, temporary disturbances in blood flow and nutrition may occur in the proximal segment, including the condyle. Further clinical and basic research is needed to clarify these mechanisms.

To date, no definitive evidence has demonstrated that mechanical loading on the condyle increases after surgery. Dynamic analyses based on CT‑ and MRI‑derived muscle measurements have shown that changes in the masseter and pterygoid muscles do not significantly increase condylar pressure or induce substantial condylar rotation [98, 99]. Additionally, postoperative occlusal force does not appear to exceed preoperative levels, even in cases where condylar height reduction is observed one year after surgery [100]. These findings suggest that overall condylar stress may not increase postoperatively. However, the distribution and direction of stress on the condyle can change significantly after surgery [43, 44, 101–103], which may be sufficient to trigger localized overload and subsequent resorption.

Simultaneous TMJ disc repositioning using the MiTek mini‑anchor system during orthognathic surgery has been reported to improve TMJ prognosis and enhance skeletal stability [14, 22, 104]. Disc repositioning may facilitate bone apposition in localized condylar regions by restoring disc coverage over the condylar head, thereby redistributing compressive forces and preventing nutritional compromise. When pathological changes are present in the TMJ, combining disc repositioning with orthognathic surgery may therefore be a reasonable and beneficial approach.

Previous studies, however, have several limitations. Sample sizes are often small, and even within the same skeletal classification or occlusal pattern, considerable individual variation exists. Evaluating occlusion, jaw morphology, and TMJ characteristics as continuous variables rather than categorical classifications may reduce bias. Additionally, many studies include predominantly female patients, limiting generalizability. Larger, standardized, multicenter studies are needed to validate these findings and further elucidate the mechanisms underlying PCR.

## Conclusion

In this narrative review, we summarized current evidence regarding changes in temporomandibular joint (TMJ) morphology before and after orthognathic surgery in skeletal Class II and Class III patients, based on CT and MRI findings, and proposed hypotheses regarding the mechanisms underlying these changes.

The clinical implications of this review are as follows. Preoperative MRI and CT imaging may be valuable for accurately evaluating TMJ morphology in patients with jaw deformities. In particular, Class II patients with anterior disc displacement and high condylar surface CT values may be at increased risk of postoperative condylar height reduction and resorption. To mitigate this risk, surgical planning may include superior repositioning of the maxillary molars via Le Fort I osteotomy to avoid excessive stretching of the pterygo‑masseteric sling, which can increase compressive loading on the TMJ. Additionally, repositioning the displaced disc to align with the direction of stress may help reduce postoperative overload. During preoperative orthodontic treatment, the use of occlusal splints to decrease condylar loading and promote metaplasia of the posterior attachment (pseudo‑disc formation) may also be beneficial.

Future studies should include larger sample sizes and standardized MRI and CT protocols to enable objective comparisons before and after surgery. Comprehensive evaluation of maxillofacial morphology—including integration of imaging modalities and physiological assessments from the same patient—is essential. Accumulation of standardized raw data across multiple institutions will facilitate more reliable and objective analyses. Our previous studies have emphasized the importance of rigorous standardization of imaging equipment and protocols to ensure reproducibility. Although meta‑analyses based on unified evaluation criteria are desirable, large‑scale multicenter studies designed to minimize bias will be critical for advancing our understanding of TMJ morphology and postoperative changes.

## Data Availability

No datasets were generated or analysed during the current study.
